# Odontoblast-like differentiation and mineral formation of pulpsphere derived cells on human root canal dentin in vitro

**DOI:** 10.1186/s13005-017-0156-y

**Published:** 2017-12-08

**Authors:** Jörg Neunzehn, Sandra Pötschke, Christian Hannig, Hans-Peter Wiesmann, Marie-Theres Weber

**Affiliations:** 1 Technische Universität Dresden, Institute of Material Science, Chair for Biomaterials, Budapester Strasse 27, D-01069 Dresden, Germany; 2 Clinic for Operative and Pediatric Dentistry, Medical Faculty Carl Gustav Carus, TU Dresden, Fetscherstraße 74, D-01307 Dresden, Germany

**Keywords:** Pulp spheres, Pulp regeneration, Odontoblast phenotype, Mineral formation, Endodontics

## Abstract

**Background:**

The revitalization or regeneration of the dental pulp is a preferable goal in current endodontic research. In this study, human dental pulp cell (DPC) spheres were applied to human root canal samples to evaluate their potential adoption for physiological tissue-like regeneration of the dental root canal by odontoblastic differentiation as well as cell-induced mineral formation.

**Methods:**

DPC were cultivated into three-dimensional cell spheres and seeded on human root canal specimens. The evaluation of sphere formation, tissue-like behavior and differentiation as well as mineral formation of the cells was carried out with the aid of optical light microscopy, immunohistochemical staining and scanning electron microscopy (SEM).

**Results:**

Spheres and cells migrated out of the spheres showed an intense cell-cell- and cell-dentin-contact with the formation of extra cellular matrix. In addition, the ingrowth of cell processes into dentinal tubules and the interaction of cell processes with the tubule walls were detected by SEM-imaging. Immunohistochemical staining of the odontoblast specific matrix proteins, dentin matrix protein-1, and dentin sialoprotein revealed an odontoblast-like cell differentiation in contact with the dentin surface. This differentiation was confirmed by SEM-imaging of cells with an odontoblast specific phenotype and cell induced mineral formation.

**Conclusions:**

The results of the present study reveal the high potential of pulp cells organized in spheres for dental tissue engineering. The odontoblast-like differentiation and the cell induced mineral formation display the possibility of a complete or partial “dentinal filling” of the root canal and the opportunity to combine this method with other current strategies.

## Background

An important and in everyday dental practice firmly established procedure is the root canal treatment. The tooth is preserved by removing the contaminated or injured dental tissue, disinfecting the canal system, and then obturating and sealing it with synthetic material. However, the treatment-related loss of the pulp and the attempt to restore it with a non-physiological substitution lead to the deprivation of pulp functions. Crucial basic functions of the pulp such as humidification and dentin formation, pain transmission, the immunological defense and the formation of tertiary dentin are eliminated by the removal of the pulp.

To improve this conventional treatment method, a further and very promising approach is the regeneration of pulp tissue using tissue specific cells (pulp stem cells or progenitor cells with stem cell characteristics) [1].

Due to the rapid progress in the fields of tissue engineering and regenerative medicine, numerous opportunities to test and implement various cell-based regeneration techniques are available. Over the past few years, stem cells derived from different dental tissues (e.g. the apical papilla, the periodontal ligament and the dental follicle) were isolated and demonstrated their potential for differentiation into different tissue cell types [2]. Moreover, the first isolation of mesenchymal stem cells from the human dental pulp of permanent teeth resulted in an increasing actuation of various international working groups in this field. Recent in vivo studies have shown that the functional recovery of pulp tissue could indeed be possible with the aid of these cells when differentiating into angiogenic, nerval or odontogenic/osteogenic cells [3–5].

The migration of pulp cells on the dentinal surface, both in vitro and in vivo, as well as the ingrowth of cell processes into dentinal tubules have been proven. With the aid of immunohistochemical staining, several studies have shown hints of different stages of odontoblast-like differentiation such as specific protein expressions, a typical odontoblastic cell shape, hints of mineral formation and histological evidences for other pulp tissue specific cell types [6, 7].

To achieve the goal of a cell-based regeneration of pulp tissue, scaffold materials (including collagen, fibrin, synthetic base materials in solid form or as hydrogels) supplemented with bioactive additives such as growth factors are widely used [8–12]. However, the usage of foreign exogeneous substances as scaffold materials is considered controversial. The unpredictable degradation of inorganic as well as organic scaffold materials indicates a risk factor concerning wound healing and complete tissue formation in vivo [13, 14].

An interesting aspect to avoid scaffolds in dental tissue engineering is the use of spheroidal micromass cultures where cell agglomerates can be applied directly, without a scaffold, into the root canal of the tooth [13, 15–17]. In medical and biomaterial research, spheres are already an established procedure when testing biomaterials as well as active substances in an in vitro cell culture system. Therefore, in the present study, DPC were extracted from human pulp tissue, cultured and transferred into scaffold-free spherical micromass cell cultures, spheres [18]. These cell agglomerates were seeded on human dentin specimens to analyze their behavior in contact with this physiological substrate by light-microscopy, immunohistochemical staining and scanning electron microscopy. Three-dimensional cell agglomerates are known to differentiate faster than 2-dimensional cell cultures without scaffolds or additives [18, 19].

In contrast to the results of earlier studies, the aim of the present investigation was not to regenerate the different components of the soft pulp tissue with their specialized functions, but rather to focus on the hard tissue formation within root canals. Throughout life a physiological age-related obliteration of the pulp chamber and root canals occurs. Hereby, a continuous and daily deposition of secondary dentin reduces the volume of the pulp. Therefore, the objective of the present study was to use this phenomenon and induce a physiological obliteration in teeth with the need of a root canal filling after an endodontic treatment. Hereby, root canals are physiologically obliterated via cell-sphere-induced biomineral formation in order to obtain a “physiological root canal-filling” inspired by the physiological age-related obliteration.

## Methods

### Human root canal preparation

In order to investigate the interactions between pulp spheres/−cells and the root canal dentin surface, human incisors, premolars and molars of donors at the age of 25–50 were extracted during routine surgical treatment. The extracted teeth underwent a root canal preparation using ProTaper (Dentsply Maillefer, Ballaigues, Switzerland) and sodium chloride 0.9% as rinsing solution to remove loose dentin particles. The roots were explored and prepared up to ProTaper finishing file F5 with a 0.5 mm diameter and a fixed taper of 5% in its apical extent. The endodontically treated roots were then cut horizontally into 3 mm thick specimens. The accumulated smear layer consisting of abrasive dust and debris inside the root canals was removed by ultrasonic desorption using 70% ethanol, 3% ethylenediaminetetraacetic acid (EDTA) and distilled water for one minute each, respectively. Finally, the prepared root canal discs were sterilised by gamma irradiation.

### Cultivation of human dental pulp cells and pulp sphere formation

Pulp tissue derived from human wisdom teeth was macerated enzymatically by the use of collagenase to isolate the cells from the surrounding tissue. The DPC were cultivated up to the fourth passage in D-MEM (low glucose), 20% FCS, 2% HEPES, 100 u/ml penicillin, 100 μg/ml streptomycin, 50 μg/ml gentamicin, 2.5 μg/ml amphotericin B (all PAA, Cölbe, Germany) at 37 °C, 5% CO_2_ and 95% humid atmosphere with a medium change twice a week.

Chambers of 96-well plates were prepared by applying 50 μl of a mixture of 20 mg/ml agarose (Biozym Scientific GmbH) in D-MEM per well to ensure a non-attachment environment for the cells. A population of 100,000 cells per well was seeded into the specifically treated cell culture dishes and incubated in the same way as the cells described above.

### Cultivating pulp spheres in a biological environment

To investigate the interaction of the pulp spheres with the root canal surface, five-day-old pulp cell spheres were transferred into the lumina of eleven human root canal specimens to be cultured. Depending on the size of the prepared root canal lumen, up to two spheres were seeded into one canal.

The sphere-seeded material was evaluated during the whole trial period by light microscopy (LM) and after 28 days by scanning electron microscopy (SEM) as well as immunohistochemical staining.

### Scanning electron microscopy

For scanning electron microscopic investigation, the cell-seeded specimens were removed from the cell culture medium, washed with phosphate buffered saline (PBS) and fixed with glutaraldehyde (4%). The samples were then dehydrated in an ascending series of isopropanol and chemically dried through a stepwise transfer into pure hexamethyldisilazane (HMDS). The dried samples were prepared for SEM-investigation and sputtered with gold-palladium. Scanning electron microscopy was carried out using a Philips ESEM XL 30 in Hi-Vacuum mode detecting backscattered electrons to investigate material contrasts as well as detecting secondary electrons for imaging.

To investigate the cell behavior directly at the dentin surface, further samples were embedded into araldite after dehydration.

Through various inter-medium levels of propylene oxide and araldite (mixing ratios of 2/1 propylene/araldite, 1/1 propylene/araldite, 1/2 propylene/araldite), the samples were transferred into pure Araldite and polymerized in silicone forms at 60 °C over a period of 3 days. The polymerized sample blocks were cut in half and were manually polished (PHOENIX ALPHA Grinder/Polisher, P240, grain size: 59 μm) to a plane in which the spheres in the root canal were detectable. A flat and smooth surface of the specimens was achieved using an ultra-microtome.

### Preparation of histological sections

The pulp sphere-seeded root canals were fixed in 3.7% neutral buffered formalin at 4 °C for 7 days, followed by demineralization in Osteosoft (Merck Millipore, Darmstadt, Germany) at room temperature for one week. After dehydration in ascending concentrations of alcohol and degreasing the samples twice in xylene, the dentin samples were embedded in paraffin. Sections of 5 μm thickness were cut by the use of a rotary microtome HM 355S (Thermo Fisher Scientific GmbH, Dreieich, Germany).

### Immunohistochemistry

Immunohistochemical staining of dentin matrix protein-1 (DMP-1) and dentin sialoprotein (DSP) was performed to evaluate the ability of pulp cells to differentiate into odontoblast-like cells. First, the samples were deparaffinized in xylene and rehydrated in a graded series of alcohol before being briefly rinsed in distilled water. The treatment with 0.1% pepsin in 0.1 mol/l HCl-solution for 20 min at room temperature uncovered the specific antigens. The samples were then washed three times with phosphate buffered saline (PBS, Sigma Aldrich Chemie GmbH, Taufkirchen, Germany) and incubated in a blocking solution (PBS, pH 7.4) containing 5% horse serum (Vector Laboratories Inc., Burlingame, USA) and 5% bovine serum albumin (BSA, Sigma Aldrich Chemie GmbH, Taufkirchen, Germany) at room temperature for 2 h to suppress non-specific binding sites. The mouse anti-human DMP-1(sc-73,633, Santa Cruz Biotechnology Inc., Heidelberg, Germany) and the goat anti-human DSP (sc-18,325, Santa Cruz Biotechnology Inc., Heidelberg, Germany) were added to a dilution of 1:50 PBS containing 0.1% horse serum and 0.1% BSA, respectively, and were incubated at 4 °C in a moist chamber overnight. After being washed three times in PBS, the bound DMP-1 antibodies reacted with the fluorescein-conjugated horse anti-mouse IgG secondary antibodies (Vector Laboratories, Inc., Burlingame, USA) while the bound DSP antibodies reacted with the Alexa Fluor 647-conjugated donkey anti-goat IgG secondary antibodies (Life Technologies GmbH, Darmstadt, Germany) at room temperature for 2 h. The nuclei of the pulp cells were stained with 4′,6-diamidino-2-phenylindole (DAPI, Life Technologies GmbH, Darmstadt, Germany). Finally, the samples were mounted with Fluoromount G (Southern Biotechnology Associates Inc., Birmingham, USA) to prevent the fading of the samples. Negative controls were obtained by substituting the primary antibodies with horse serum and goat serum. All images were acquired with an epifluorescence microscope (Axioskop II, ZEISS, Oberkochen, Germany).

## Results

In the present study, a physiological interaction between DPC and the human dentin surface was revealed by scanning electron microscopy, and an odontoblastic differentiation of human pulp cell spheres was proven by immunohistochemical staining of DMP-1 and DSP. Furthermore, for the first time scanning electron microscopic investigation of the sphere-seeded root canals confirmed an odontoblast-like phenotype of the cells that grew out of the spheres. In addition, a strong cell-induced mineral formation could be detected as well.

### Cell-cell and cell-dentin interaction

**Fig. 1:**
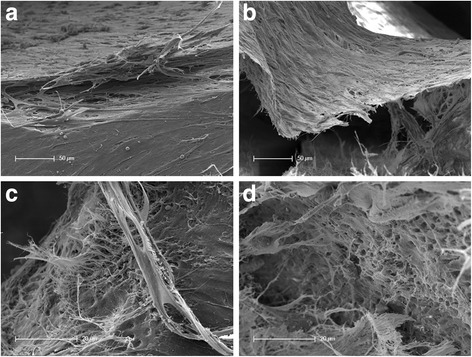
SEM-investigation of cell-cell and cell-dentin interactions in human root canals after 28 days of cultivation. **a**. Multilayered cell stack/ layer with tight cell-cell contacts on the dentinal surface. **b**. Sturdy cell layer after detaching of the cell accumulation from the root canal wall. **c**. Cell matrix filaments connected to root canal dentin after detachment of superimposed cell layers. **d**. Replicated dentin structures from cell matrix on root canal dentin

When investigating the cells that grew out of the spheres by scanning electron microscopy, a close cell-cell contact and a cell-dentin contact were visible (Fig. 1). The migrated cells aligned themselves in multilayers on the biological dentin surface. Especially in areas of the samples where the cell layers were separated from the dentin surface due to artificial drying and preparation, a very close bond between the cells forming a solid cell layer was detected. In addition, an intensive cell-dentin contact could also be revealed in the areas of the root dentin where the cell layers had been detached. On the exposed dentin surfaces, fibers of extracellular matrix from the torn off cell layers extended into the root canal lumen (Fig. 1b, c). Alongside these fibers, the formation of small lumina within the extracellular matrix which imitate the shape and form of small dentinal tubules in the root dentin was identified (Fig. 1c, d).

**Fig. 2:**
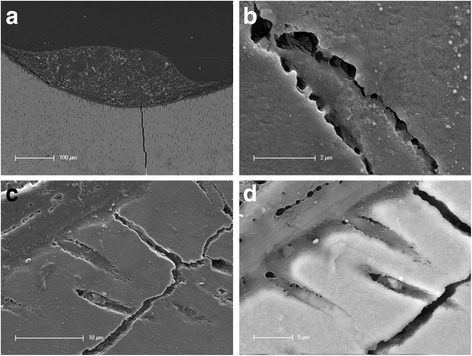
SEM-investigation of the ingrowth of cells from spheres into tubules after 28 d of cultivation. **a**. Overview of the sample cut vertically - sphere is located on root dentin surface. **b**. Migrated cell processes into a dentinal tubule with direct contact to the surrounding dentin. **c**. Grown in cell processes from the cell layer of the sphere into the mineralized dentin layer of the root canal; topographical contrast. **d**. Grown in cell processes from the cell layer of the sphere into the mineralized dentin layer of the root canal; backscattered electron contrast (“material contrast”)

Further insight concerning the interaction between cells inside a sphere was realized by sectioning a pulp sphere placed in a human root canal that had been embedded in araldite after cultivation (Fig. 2a). Using appropriate magnification of the interface between the sphere and the root canal dentin, the ingrowth of cell processes of the sphere cell layer into dentinal tubules of the root canal was detectable (Fig. 2b-d).

These cellular processes interacted through small extensions with the walls of the dentinal tubules (Fig. 2b). Figure 2c and d show the ingrowth of cell processes from the cells belonging to the sphere into the dentinal tubules of the mineralized dentin layer based on SE-Detection and RE-Detection (in Fig. 2d, the mineralized dentin appears brighter through the backscattered electron contrast, “material contrast”).

### Odontoblast specific protein expression

The dentin matrix protein-1 (DMP-1) and dentin sialoprotein (DSP) are relevant key proteins for the differentiation of pulp cells into odontoblasts. In addition, both proteins play an important role in the mineralization process of dentin.

**Fig. 3:**
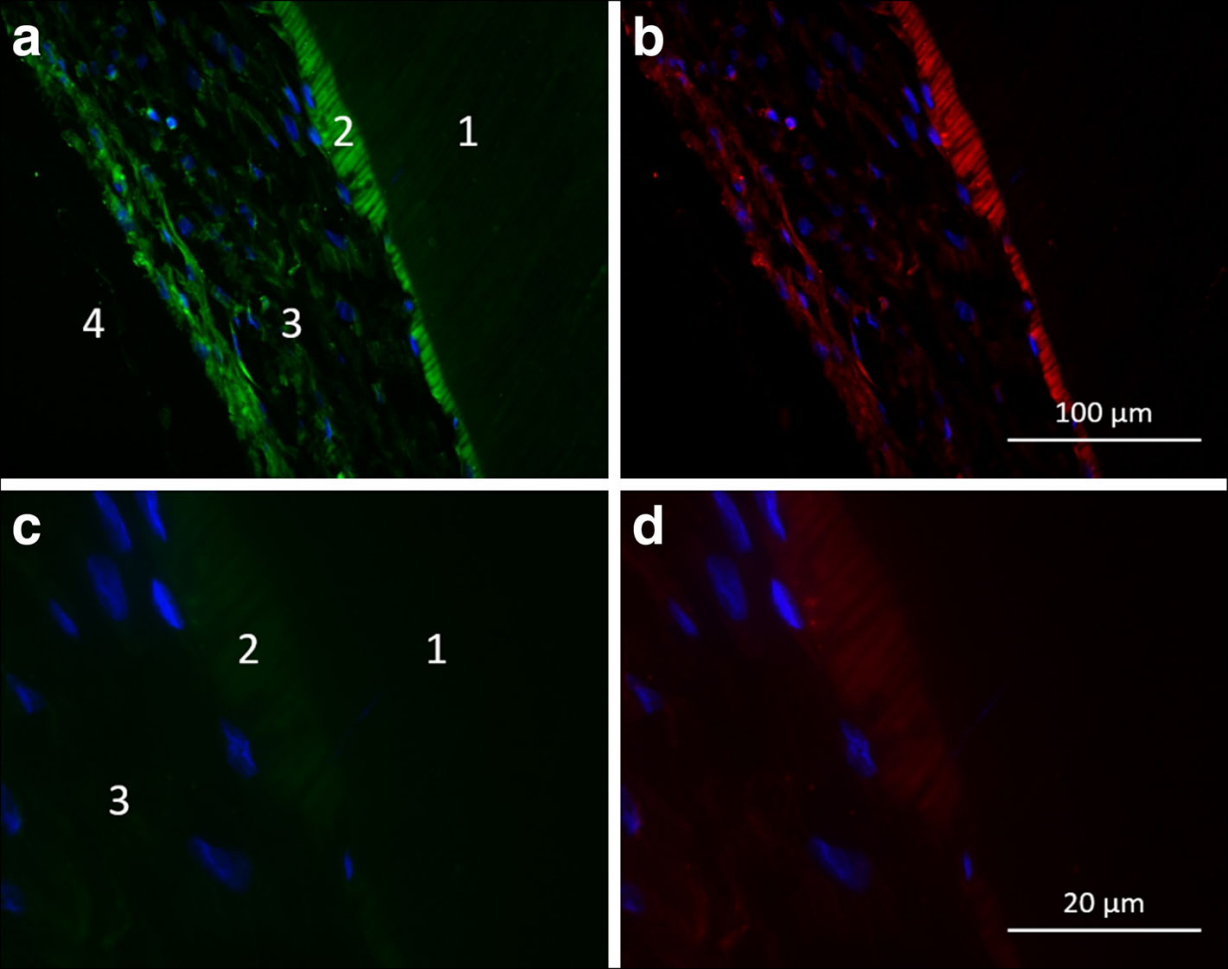
Fluorescence microscopic analysis of spheres in human root canals. The histological sections are divided into the areas (1) root canal dentin, (2) influenced area by EDTA, (3) cellular agglomerate of the sphere and (4) root canal lumen. In the different paraffin sections, the nuclei of the pulp cells are stained “blue”, the dentin matrix protein-1 (DMP-1) “green” and the dentin sialoprotein (DSP) “red”. **a**. and **b**. In the samples treated with the EDTA, a uniform layer (2) is visible in which the two labeled proteins are apparently released and detectable. The two odontoblast-specific proteins are also clearly detectable in the cell layers of the sphere (3). The cells within the spheres are evenly distributed (blue-colored cell nuclei) within the spheres. **c**. and **d**. In the transition region between region 2 and 3, the cells lie flat with elongated nuclei on the root canal dentin surface

**Fig. 4:**
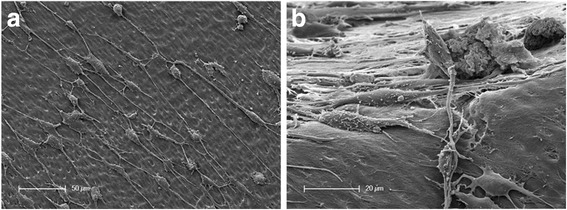
Scanning electron microscopical investigation of the phenotype of single, mature and sphere-derived cells. **a**. Dentinal root canal surface covered with odontoblast-like cells with small cell bodies and strongly extended cell processes. **b**. Cells with odontoblast-like, elongated cell bodies and extended cell processes at the junction of root canal dentin and root canal lumen

Therefore, the expression of the odontoblastic differentiation markers DMP-1 and DSP was examined by immunohistochemical staining. Furthermore, this approach allowed the investigation of the interactions between cells and dentin after the dentin underwent a treatment with EDTA. Fluorescence microscopical analysis of the sphere-seeded root canals yielded a distinct differentiation of odontoblasts. As shown in Fig. 3, odontoblastic markers DMP-1 (green) and DSP (red) were highly expressed in the cells of the sphere confirming the existence of mature odontoblastic cells. The nuclei of the cells within the spheres were labeled with DAPI (blue) to observe the cell arrangement. The examination revealed a uniform distribution of the cells. In the outer zone of the sphere, the cells lay flat on the dentin and the elongated shape of the cells’ nuclei showed the typical morphology of the nuclei of odontoblasts. The highest amount of positive DMP-1 and DSP signals was detected in the outer zone of the spheres, indicating that both proteins play an important role in the mineralization process of dentin. The typical odontoblast-like characteristics could not be revealed among the cells that grew out of the spheres by immunohistochemical staining. However, using scanning electron microscopy, the formation of long cell processes equivalent to the odontoblastic phenotype could be detected among the cells that grew out of the spheres and migrated over the dentin surface of the root canal (Fig. 4). Furthermore, cell processes similar to Tomes’ fibers grew into the dentinal tubules (Fig. 2). Figure 4a shows a root dentin surface with apparent dentinal tubules. The surface is covered with cellular matrix. The adherent elongated cells exhibit very short cell bodies compared with the threadlike cell processes (in Fig. 4a up to 150 μm long). Figure 4b shows cells of an odontoblastic phenotype on the dentin surface of the root canal lumen at a higher magnification. A cell with a columnar, cylindrical cell body is shown that is directly connected with small thin filamentous processes to the cell body of an adjacent cell. This interaction between different cells of this prominent morphology can be noticed among nearly all investigated cells on the dentinal surface (Fig. 4a, b).

**Fig. 5:**
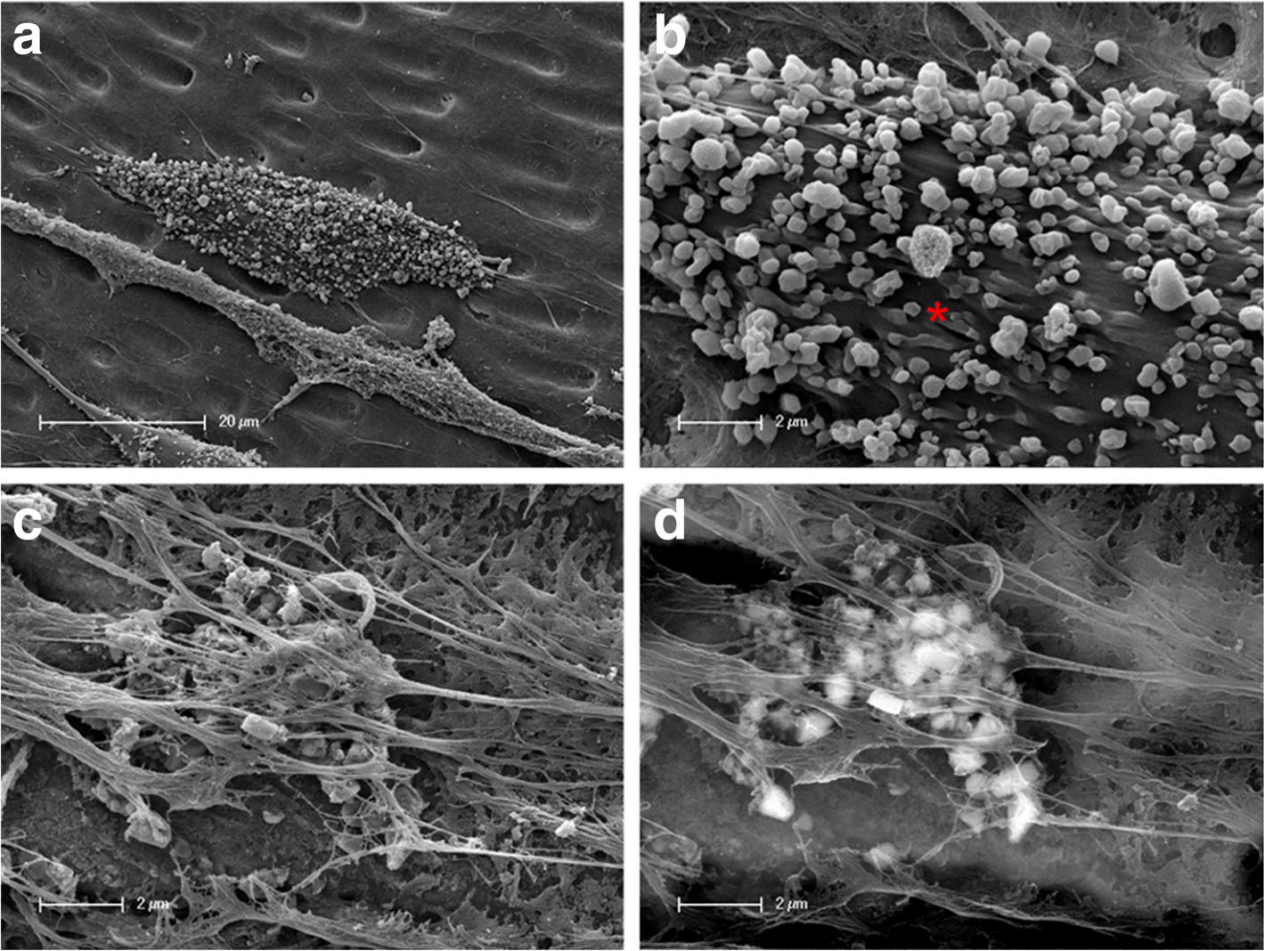
Different mineralization mechanisms. **a**. and **b**. Surface of a cell with mineral particles differing in form and size as well as vesicle constrictions of minerals being transported from the inside of the cell to the surface (*). **c**. and **d**. Cell-induced mineral formation within a multiple cell unit; **c** topographic contrast, **d** backscattered electron contrast (“material contrast”)

Another phenomenon that could be detected by scanning electron microscopy is the fact that the mature cells that migrated away from the sphere formed a considerable amount of mineral deposits. This interesting phenomenon was expressed in two different forms. First, cells displayed several round and square minerals on the surface of their cell bodies (Fig. 5a, b). Moreover, cellular constrictions were observed on the surface of the cell bodies containing minerals that were transported from the inside of the cell to the surface (Fig. 5b).

Secondly, a mineralization process could be detected by scanning electron microscopy using the topographical contrast (Fig. 5c) as well as the backscattered electron contrast (“material contrast”) (Fig. 5d). As a result, several cells and cell processes superimposed over each other on the dentin of the root canal so that cell-induced mineral formation within a cell unit could take place. The comparison between the scanning electron images (Fig. 5c, d) shows that mineral particles in various forms and sizes were located beneath and within the superimposed cell material (Fig. 5d) as well as on the surface of the cell unit (Fig. 5c).

## Discussion

In the present study, the odontoblastic phenotype of differentiated odontoblast-like cells could be visualized using SEM for the first time.

For this purpose, DPC were cultivated into spheres and seeded onto human dentin specimens. A very interesting aspect is that DPC seem to keep their stem cell character even after being fused into three-dimensional cell spheres [20, 21]. In this study, the profound cell contact within the spheres led to a subsequent pre-differentiation of the DPC and had a major influence on the differentiation process into odontoblast-like cells. Furthermore, the direct contact of the cells that grew out of the spheres with the human root dentin surface was trigger enough for the cells to differentiate into odontoblast-like cells. Subsequently, the odontoblast-like cells stimulated a bio-mineral formation most likely initiated by the human root dentin surface and its containing dentin matrix proteins since no differentiation or mineralization factors were added. These findings might be the base for a new and biological root canal filling strategy.

In certain circumstances, mainly caused by direct stress on teeth (trauma, caries), a physiological obliteration of the root canal occurs in vivo. It is mainly characterized by the deposition of hard tissue within the root canal. The exact mechanism of root canal obliteration is not yet known; however, it is believed to be related to the damage of the neurovascular supply of the pulp at the time of injury [22–25].

Furthermore, it is known that in the case of odontoblast damage, a calciotraumatic reaction occurs which leads to the formation of tertiary dentin. The tertiary dentin can be formed by original odontoblasts as a response to attrition, caries or other stimuli (reactionary dentin) or due to odontoblast damage such as odontoblast aspiration and trauma by newly differentiated odontoblast-like cells (reparative dentin) [26]. It is believed that undifferentiated ectomesenchymal cells already present in the pulp, for example in the subodontoblastic layer, differentiate into newly formed odontoblast-like cells and produce reparative dentin [27].

Also non-tubular fibrodentin, osteodentin, has been identified within pulp chambers and root canals. Due to its regular shape and uncalcified border, it is suggested that orthodentin is deposited by a hard tissue-forming cell which has not yet been identified.

The loss of the nervous and vascular system after a root canal preparation leads to the idea that DPC, prediffierentiated through the intense cell-contact within the spheres and differentiated into odontoblast-like cells, could behave similar to odontoblasts in stress conditions or newly formed odontoblast-like cells from undifferentiated ectomesenchymal cells due to odontoblast damage. A purposely induced and oriented obliteration via the application of dental stem cell spheres onto the root dentin surface could possibly serve as a biological and semiphysiological root canal filling in itself instead of using synthetic root canal filling materials such as gutta-percha. In the clinic, the spheres would be applied directly into the prepared and disinfected root canal. Unlike synthetic root canal filling materials, DPC can differentiate into odontoblast-like cells. Moreover, the processes of these cells are able to migrate into the ramifications and dentinal tubules of the root canal system (Fig. 2). However, it is necessary that the root canal is treated with an irrigation protocol which still has not been completely established. Literature describes that DPSC would not attach to dentin that had been treated with NaOCl before the DPSC were applied [28]. Further, the influence of root canal disinfectants on growth factor release from dentin is a very important factor. The effect of growth factors on pulpal stem cell migration, proliferation and differentiation could be beneficial for regenerative therapies. EDTA for example releases the highest amount of growth factors compared to chlorhexidine and sodium hypochloride. Yet, a treatment with chlorhexidine prior to EDTA irrigation increases the growth factor release from dentin whereas sodium hypochloride decreases the growth factor release [29].

Furthermore, the mineral produced by the odontoblast-like cells could seal and eventually obliterate the prepared and disinfected root canal system. Synthetic root canal filling materials still lack the complete access to all areas of the cleaned and prepared root canal [30–32].

Endodontically prepared root canals lack the blood and nervous system supply. However, odontoblasts and presumably odontoblast-like cells contain mechano-thermosensitive transient receptor potential ion channels (e.g. TRPV1–4, TRPA8, Kca, PC1, PC2) located on the odontoblastic membrane and at the base of the cilium [33]. Therefore, a “biological root canal filling” containing these cells would possibly be able to sense to some extent temperature variations, movements and pressure [20, 21]. In the case of complete obliteration, the root would be stabilized by bio-minerals.

Previous studies indicated that the synthesis and expression of odontoblast matrix protein-1 (DMP-1) and dentin sialoprotein (DSP), the formation of extracellular matrix and the subsequent mineralization as well as the expression of a distinct phenotype are cell-specific characteristics for odontoblasts [34–38].

In this in vitro investigation, immunohistochemical analysis indicated the expression of dentin matrix protein-1 and dentin sialoprotein within the cells of the spheres yielding odotoblastic differentiation (Fig. 3). Furthermore, the results of the immunohistochemical investigations revealed a direct contact between the cells of the sphere and the root canal surface.

After the formation and expression of specific matrix proteins, the next physiological differentiation step of odontoblasts is the formation of extracellular matrix (ECM) that will subsequently mineralize. In the present study, the cell-induced mineral formation, as well as the matrix formation, was detected via scanning electron microscopy. ECM formation took place among cell layers that coated the root canal samples which were tightly intergrown with the dentinal surface (Fig. 1). In addition, matrix formation occurred among the cells which grew out of the spheres and induced a reproduction of physiological dentinal structures. The tight connection between the cell layers and the dentinal surface shows the compatibility between both components and indicates a close physiological interaction between the cells and the dentin (Fig. 1). Moreover, the structure-oriented migration of the cells when growing out of the spheres and the ingrowth of the cells into the dentinal tubules emphasize the close bond between the root dentin and the cells. Growth factors such as TGF-β (transforming growth factor-β) that can evidently be released from the dentinal surface could be the reason for the cell-induced dentinal structure formation and therefore support the physiological odontoblastic differentiation without additional growth factors [39]. However, in order to optimize the differentiation of the cells into odontoblasts or odontoblast-like cells, a pre-differentiation of the cells inside the spheres and a potential calcium ion release from the dentin could be beneficial [40].

A further proof for odontoblastic differentiation is the cell-induced ability of the differentiated odontoblasts or odontoblast-like cells to perform mineral formation and eventually generate dentin. The ability to mineralize is an essential phase in the cell-induced dentin formation process which can only be performed by odontoblasts and which indicates a physiological development of the cells [27, 41].

In the cell culture trials of the present study as well as previous findings [18], mineral formation inside the spheres as well as on the surface of the cells that migrated out of the spheres was clearly visible using scanning electron microscopy. Inside the spheres, mineral particles could be clearly distinguished from the surrounding extracellular matrix by means of backscattered electron contrast (“material contrast”) [18]. The morphology as well as the detected vesicular constrictions of mineral particles from the cells in the present study is comparable with the origin and form of cellular-induced minerals regarding the odontoblastic mineralization front in vivo [42]. Previous findings regarding mineral formation related with odontoblasts were mostly visualized using histological, immunohistochemical and light microscopic analysis [43]. Moreover, evidence whether an odontoblastic or osteogenic differentiation had occurred was missing or was solely discussed [44, 45].

In the present study, the cells were spread out flat on the mineral of the dentin and appeared to have elongated cell nuclei, small cell bodies and long cellular processes (Fig. 3). Neuronal cells appear to have a similar phenotypic expression and their cell processes grow into dentinal tubules as well, though neuronal cells do not express mineral deposits on their cell bodies as detected on the odontoblast-like cells of the present study. For the first time, odontoblastic differentiation was verified by the visualization of the corresponding phenotype and the varying differentiation phases via scanning electron microscopy alongside electron-optical and immunohistochemical analysis.

## Conclusion

The results of the present study reveal the high potential of DPC, especially in form of spheres, for dental tissue engineering. An odontoblastic phenotype was verified using scanning electron microscopy after the differentiation of dental stem cells into odontoblast-like cells. Furthermore, biomineral formation was possible when placing three-dimensional cell-spheres on root dentin without the addition of growth factors.

The formation of biomineral similar to dentin by odontoblast-like cells displays the possibility for the base of a new and biological root canal filling strategy. In addition, this method could possibly be combined with other current strategies that revascularize and revitalize treated root canals.

## Abbreviations

DAPI: 4′,6-diamidino-2-phenylindole
D-MEM: Dulbecco Modified Eagle Medium
DMP: Dentin matrix protein
DPC: Dental pulp cell
DSP: Dentin sialoprotein
ECM: Extra cellular matrix
EDTA: Eethylenediaminetetraacetic acid
FCS: Fetal calf serum
HCl: Hypochloric acid
HMDS: Hexamethyldisilazane
IgG: Immunoglobulin G
LM: Light microscopy
PBS: Phosphate buffered saline
SEM: Scanning electron microscopy
